# The metabolomics of human aging: Advances, challenges, and opportunities

**DOI:** 10.1126/sciadv.add6155

**Published:** 2022-10-19

**Authors:** Daniel J. Panyard, Bing Yu, Michael P. Snyder

**Affiliations:** ^1^ Department of Genetics, Stanford University School of Medicine, Stanford University, Stanford, CA 94305, USA.; ^2^ Department of Epidemiology, Human Genetics, and Environmental Sciences, School of Public Health, The University of Texas Health Science Center at Houston, Houston, TX 77030, USA.

## Abstract

As the global population becomes older, understanding the impact of aging on health and disease becomes paramount. Recent advancements in multiomic technology have allowed for the high-throughput molecular characterization of aging at the population level. Metabolomics studies that analyze the small molecules in the body can provide biological information across a diversity of aging processes. Here, we review the growing body of population-scale metabolomics research on aging in humans, identifying the major trends in the field, implicated biological pathways, and how these pathways relate to health and aging. We conclude by assessing the main challenges in the research to date, opportunities for advancing the field, and the outlook for precision health applications.

## Teaser

This review describes the state of human aging metabolomics research in population studies.

## INTRODUCTION

Aging is a fundamental part of the human experience, and it has long been understood to be a crucial component of health and disease. Population estimates of mortality fundamentally incorporate and adjust for age, which is widely considered the most important predictor of mortality (*1*) and can be seen in practice as early as Edmond Halley’s age-specific estimates of mortality in 1693 (*2*). The centrality of aging in health largely stems from the age-related increase in risk for many diseases. Cancer, cardiovascular disease, neurodegeneration, and many other common disorders are more common among the elderly than among the young (*3*). Defining the biology that drives aging is challenging, but theories of aging have coalesced around several key hallmarks, ranging from cellular senescence and stem cell exhaustion to mitochondrial, proteostasis, and genomic dysfunction (*4*). As the world’s population ages, with one in six expected to be 60 or older by the end of 2030 (*5*), understanding these physiological pathways and how to intervene in them will be critical to the prevention and management of the major drivers of morbidity and mortality.

In recent decades, technological advancements have opened up new possibilities for obtaining molecular data at a population scale. One of these technologies is metabolomics, which refers to the study of small molecules in the body, generally less than 1500 Da in mass (*6*–*9*). The Recon3D resource (*10*) has mapped over 4000 unique metabolites in a model of human metabolism, comprising over 13,000 metabolic reactions, and the Human Metabolome Database (HMDB) (*11*) has annotated over 200,000 metabolites that may potentially be found in humans, including both endogenous (synthesized or generated by the body) and exogenous (derived from the environment) (*7*) molecules. Metabolites span a diversity of physiological processes, including the building blocks of the major macromolecules [e.g., amino acids, nucleic acids, carbohydrates, and fatty acids (FAs)], functional nutrients (e.g., vitamins and cofactors), and compounds such as sex hormones, drug intermediates, and toxins. While this molecular diversity makes chemical identification more challenging (*6*), it also makes the metabolome an attractive dataset for application to many biomedical problems (*12*, *13*), such as diabetes and insulin resistance (*14*, *15*), cancer (*16*), atherosclerosis (*17*), and Alzheimer’s disease (*18*, *19*). Since pharmacological interventions are often small chemical substances themselves, metabolomics is of particular interest to pharmacologic studies looking to identify potential drugs or treatment targets (*9*).

The decreasing cost and increasing scalability of metabolomics platforms have led to a proliferation of cohorts and biobanks adding metabolomics to their studies. For instance, the U.K. Biobank (*20*), one of the largest population cohorts to date, announced a project in 2018 to measure over 200 metabolites in half a million blood samples (*21*); the Trans-Omics for Precision Medicine (TOPMed) program has funded metabolomics collection in over 60,000 samples from diverse populations to pair with deep phenotyping and whole-genome sequencing data (*22*); and the Consortium of Metabolomics Studies (COMETS) has been working since 2014 to combine blood metabolomics data from dozens of cohorts worldwide for large-scale biomedical research (*23*). These studies represent a new era of population health research and molecular epidemiology that has enabled an unprecedented molecular view of aging processes with profound implications for precision health applications. In this review, we summarize the current state of population metabolomics research in aging in human cohorts, evidence for population-specific effects, the biological themes that have emerged among these studies and how they relate to health and aging metabolism, major challenges in the field, and opportunities for novel aging research and precision health going forward.

## HISTORICAL TRENDS IN POPULATION AGING METABOLOMICS RESEARCH

**Fig. 1.**
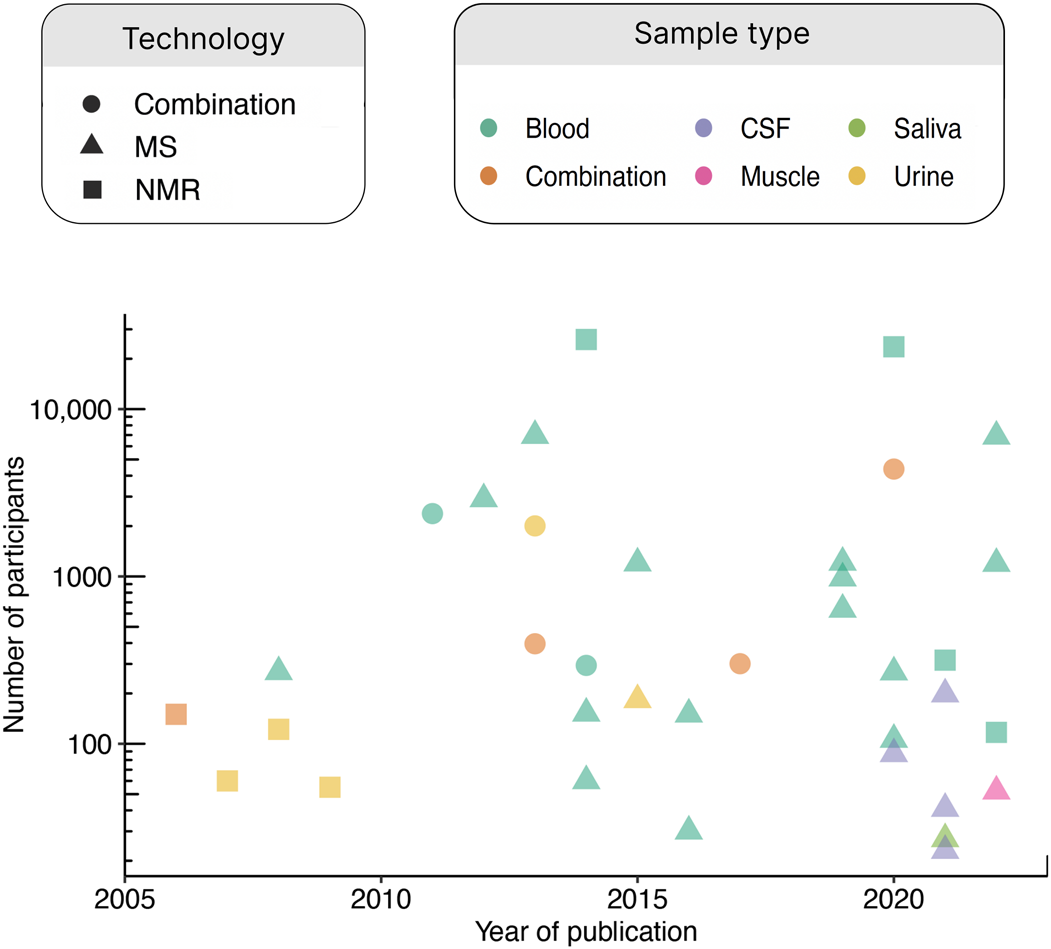
Timeline of human aging metabolomics population studies. A timeline showing the sample size of aging metabolomics studies (by number of participants on a logarithmic scale) in humans is shown, including the metabolomics technology used and the sample type. When multiple technologies or sample types were used, the study is considered a “combination” study. There has been no strong trend in sample size over time; instead, the field is dominated by occasional studies with much larger sample sizes than the rest. There has been a shift toward more MS-based technologies, although NMR studies are still being conducted and published. Most studies have used blood or urine, although in the 2020s, a greater diversity of sample types (namely, CSF, saliva, and muscle) have started to be seen in the literature.

There is a long history of studies of how metabolism changes with aging, from early animal studies of longevity to cellular and molecular studies of aging processes. With the advent of metabolomics technologies that can process samples at a large scale and reasonable cost, human population studies of aging with metabolomics data began to emerge in the late 2000s. Since then, several dozen such studies have been published with a variety of populations, age ranges, sample types, study designs, and underlying metabolomics technologies (Fig. 1 and Table 1) (*24*–*59*). The number of participants has reached the thousands in several studies [including two studies with 20,000 or more participants (*34*, *48*)], a feat that has become more common with the presence of ongoing, population-scale cohorts with biobanked samples and commercially available platforms for standardized metabolite quantification.

**Table 1.** Summary of human population studies of the metabolome and aging.

**Year**	**First author**	Region*	**Participants†**	**Sample source**	**Technology**	**Analytes**
2006	Kochhar	Europe	150	Urine and plasma	NMR	256 (urine) and 128 (plasma) variables
2007	Slupsky	North America	60	Urine	NMR	Binned spectra and 50 metabolites (targeted)
2008	Lawton	North America	269	Plasma	MS	>300 metabolites
2008	Psihogios	Europe	122	Urine	NMR	194 spectral regions
2009	Gu	North America	55	Urine	NMR	800 frequency bins
2011	Vaarhorst	Europe	2375	Serum	NMR and others	7 lipoprotein and other lipid-related measurements
2012	Yu	Europe	2904	Serum	MS	163 metabolites
2013	Menni	Europe	6942	Serum and plasma	MS	280 (serum) and 456 (plasma) metabolites
2013	Swann	Multiple	2005	Urine	NMR and MS	64,000 spectral variables (NMR)
2013	Collino	Europe	396	Serum and urine	NMR and MS	163 metabolites (MS), 63 eicosanoids (MS), and 12,000 spectral variables (NMR)
2014	Auro	Europe	26,065	Serum and plasma	NMR	135 metabolites
2014	Montoliu	Europe	294	Serum	NMR and MS	12,000 spectral variables (NMR) and 174 lipids (MS)
2014	Lee	East Asia	152	Plasma	MS	~2000 variables
2014	Saito	‡	60	Serum and plasma	MS	297 metabolites
2015	Thévenot	Europe	183	Urine	MS	170 metabolites
2015	Dunn	Europe	1200	Serum	MS	4584 metabolite features
2016	Jové	Europe	150	Plasma	MS	2678 metabolite features
2016	Chaleckis	East Asia	30	RBCs	MS	126 metabolites
2017	Rist	Europe	301	Plasma and urine	NMR and MS	442 (plasma) and 531 (urine) analytes
2019	Chak	Europe	976	Serum	MS	122 metabolites
2019	Darst	North America	1212	Plasma	MS	1097 metabolites
2019	Johnson	North America	635	Plasma	MS	360 metabolites
2020	Ahadi	North America	106	Plasma	MS	722 metabolites
2020	Bunning	North America	268	Plasma	MS	770 metabolites
2020	Robinson	Europe	4,383	Serum and urine	NMR and MS	28,941 metabolite features used in age prediction models
2020	Peters	Europe	87	CSF	MS	8036 metabolite features (untargeted) and 206 metabolites (targeted)
2020	van den Akker	Europe	23,590	Serum	NMR	62 metabolites analyzed of 226 metabolites available
2021	Hwangbo	North America	198	CSF	MS	7697 metabolite and 1070 lipid features
2021	Mallol	Multiple	316	Plasma	NMR	173 metabolite features
2021	Peters	Europe	41	CSF	MS	1841 metabolite features
2021	Teruya	East Asia	27	Saliva	MS	99 metabolites
2021	Carlsson	Europe	23	CSF	MS	70 metabolites
2022	Watanabe	East Asia	1193	Plasma	MS	94 metabolites
2022	Janssens	Europe	52	Muscle	MS	137 metabolites
2022	Verri Hernandes	Europe	6872	Serum	MS	175 metabolites
2022	Li	East Asia	117	Plasma	NMR	35 identified metabolites

*Refers to the study population.

†Includes replication samples when applicable.

‡Participants were described as “healthy Caucasian volunteers.”

While there is a great and ever-increasing variety of technologies used to generate metabolomics data at scale, most studies may be categorized as using either nuclear magnetic resonance (NMR) or mass spectrometry (MS) to identify and quantify metabolites, with NMR being more common in the earlier studies (~2005–2010) and MS being the dominant technique since. A number of important differences exist between NMR- and MS-derived metabolomics data (*7*–*9*), including a difference in the kinds of metabolites typically captured. NMR platforms tend to capture larger structures such as lipoproteins in great detail (*46*) (hence the presence of many variables related to lipoprotein particle size and density) while not generally capturing as much of a variety as MS platforms (*60*). One complication is that the unit of analysis has varied over time and by methodology. While modern commercial platforms typically provide values for specific metabolites (although sometimes unidentified), older iterations of both NMR and MS metabolomics would sometimes be analyzed as features from the raw data. Some papers thus analyze “metabolite features” or “spectral variables” that may number in the thousands or tens of thousands, yet these features may not necessarily all be mapped to corresponding metabolites by the authors except for particular findings of interest. Direct comparisons of the number of metabolites analyzed across the history of the field, then, are difficult to make.

As metabolomics technology has matured, the biological context studied by aging metabolomics researchers has diversified. While earlier studies primarily focused on plasma, serum, and urine samples, studies in the 2020s have expanded to more specialized contexts such as cerebrospinal fluid (CSF) (*47*, *49*, *51*, *53*), saliva (*52*), and muscle (*55*). The biology driving the metabolomic signatures of these sample contexts can differ substantially, leading to unique insights as described below.

Several resources have been developed to organize the major findings of aging metabolomics studies, including curated lists of metabolites associated with age and their direction of effect in online databases such as HMDB (*61*) and MetaboAge (*62*). Several reviews have also been published that have covered particular studies, metabolites, and general trends identified in the aging metabolome (*63*, *64*).

## METABOLOMIC PATHWAYS IMPLICATED IN HUMAN AGING

**Fig. 2.**
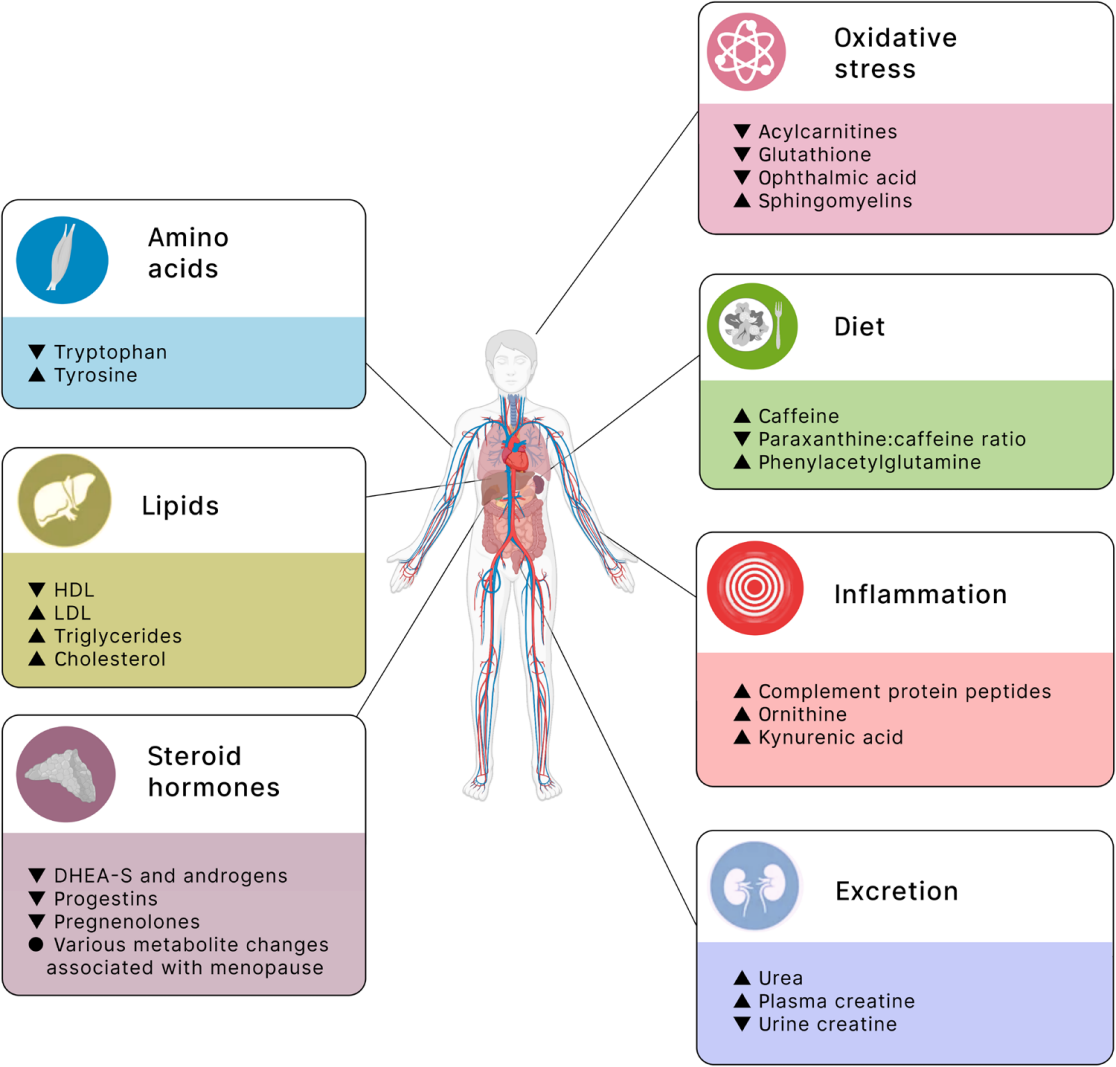
Overview of metabolomic associations with aging. An overview of the major metabolite changes associated with aging in human cohort studies is provided. The changes are grouped into seven major categories: lipids, steroid hormones, excretion, amino acids, diet, oxidative stress, and inflammation. These themes are broadly grouped by chemical structure (themes on the left) and biological pathway (themes on the right). Within each theme are some of the most consistently observed changes, with the change with age noted as an up (increased with older age) or down (decreased with older age) arrowhead. Some of the icons used in this figure were created with BioRender.com.

Despite changes in the scale, technology, and sampling space studied, a number of themes have emerged regarding both the individual metabolites and metabolic pathways associated with aging in human population studies (Fig. 2). Below, we summarize these findings into seven major pathways of metabolomic aging: lipids and lipoproteins, steroid hormones, the renal system and excretion, amino acids and muscle, diet, oxidative stress, and inflammation.

### Lipids and lipoproteins

One of the most readily apparent metabolic pathways associated with age (and one that is well known outside of the metabolomics field) involves lipids and lipoproteins, such as very-low-density lipoprotein (VLDL), low-density lipoprotein (LDL), high-density lipoprotein (HDL), triglycerides (TGs), cholesterol, FAs, and polyunsaturated FAs (PUFAs). Older individuals tend to have lipid profiles that are considered worse clinically, such as lower HDL and higher VLDL, LDL, TGs, cholesterol, and FAs. With NMR platforms providing good detail on these metabolites, these associations were captured early on in NMR studies of both blood and urine. For example, Kochhar *et al.* (*24*) observed higher total lipoproteins and lipids in older men. Vaarhorst *et al.* (*29*) observed larger LDL particles and lower TGs among the children of nonagenarians, who presumably have a better lifespan, than these children’s partners, who were the controls of the study. Auro *et al.* (*34*) observed increased VLDL, LDL, cholesterol, and TG with age, changes that also differed by sex in terms of timing. These lipids tended to increase starting around the 30s for men but not until around age 50 for women, a finding the authors suggested might be driven by the timing of menopause for women. While VLDL typically increases with age, Li *et al.* (*58*) found that VLDL was lower among long-lived groups, including centenarians and nonagenarians, compared to a reference elderly population aged 60 to 89. MS-based studies have identified similar changes as these studies, including higher cholesterol associated with age in women (*40*), higher PUFAs with age (*45*), and a general implication of altered lipids and lipid metabolism with aging (*38*).

### Steroid hormones and menopause

Another well-established metabolomic association with age is with steroid hormones and menopause. Several studies have observed a general decrease in steroid hormones in human cohorts, including decreases in dehydroepiandrosterone sulfate (DHEA-S; an androgen) (*26*, *57*) and in androgens, progestins, and pregnenolones (*42*). Studies that built models to predict age based on metabolomics have noted that DHEA-S and other steroid hormones (androsterone and progesterone derivatives) were included as predictors (*31*, *45*). Many metabolomic changes have also been observed that are presumably related to the onset of menopause, including an increase in sphingomyelins, certain amino acids (such as glutamine, tyrosine, and isoleucine), and changes in lipids and lipoproteins (*34*). A Japanese study (*54*) designed to identify blood metabolite changes with menopause identified numerous changes, particularly related to the tricarboxylic acid (TCA) cycle, urea cycle, and homocysteine metabolism. In a study of blood and urine metabolites in a German cohort, researchers found that menopause status could be predicted with 85 to 88% accuracy from metabolites, a finding supported by their general observation of increases in many metabolites in women around the age of 50 (*40*).

### Renal system and excretion

Many studies have also implicated the renal system and excretion. Researchers have noted altered levels of several metabolites as potentially indicative of changes in kidney function (declining with age) or urea metabolism, including aging-related increases in urea (blood) (*26*), ornithine (blood) (*26*), trimethylamine *N*-oxide (TMAO; urine and plasma) (*27*, *58*), glutamine (urine) (*35*), citrulline [red blood cells (RBCs)] (*39*), pantothenate (RBCs) (*39*), dimethyl guanosine (RBCs) (*39*), and *N*-acetyl-arginine (RBCs) (*39*) and decreases in β-hydroxy-β-methylbutyrate (HMB; urine) (*32*) and 1,5-anhydroglucitol (RBCs) (*39*). Studies have also found an enrichment of urea cycle metabolites in metabolomic clock models (*46*) and metabolites associated with menopause (*54*). Creatine and creatinine levels have also commonly been associated with aging, which, some researchers have argued, could be indicative of altered kidney function (*32*, *40*, *44*, *45*). Creatine levels have been reported to increase with age in plasma (*26*) and decrease in urine (*32*), while creatinine levels have commonly been associated with age (*31*, *40*, *45*), typically increasing with age in plasma (*44*, *45*) and decreasing with age in urine (*25*, *27*, *40*) and saliva (*52*), although with some exceptions among putatively healthier agers and centenarians (*35*, *44*) and among children where urine creatinine levels increased with age (*28*, *45*).

### Amino acids

Changes in amino acid levels with age have also been observed, although the trends are more difficult to summarize. Broadly speaking, amino acids tend to change with age but with varying direction. Two studies in plasma found most amino acids increased with age (*26*, *42*), leading some to theorize that this broad increase may be due to increased protein and amino acid catabolism (*24*, *26*). In contrast, one study in serum found that most amino acids gradually decreased with age (*30*). The difference in direction of effect might be due to the difference in sample type or the specific amino acids included, particularly since amino acids encompass a variety of molecules, so focusing on specific amino acids might be more interpretable. As an example, a study in serum samples found tryptophan, threonine, serine, methionine, and cysteine decreasing with age, while tyrosine increased (*37*). Two of the more consistently reported changes in amino acids include a decrease with age in tryptophan in both plasma (*40*) and serum (*30*, *33*, *37*) and an increase in tyrosine in serum (*34*, *37*, *41*) and plasma (*24*, *40*). In metabolomic-based models of age, amino acids have been effective predictors, including one study where l-methionine increased among individuals who had a faster rate of biological aging (*43*). A study in saliva found citrulline, the amino acid–derived carnitine, and glutamate to be associated with age, the last of which was thought to reflect taste-related processes (*52*). Last, the changes in creatine and creatinine levels described above may also be driven by changes in muscle tissue turnover or changing muscle mass throughout life (increasing through childhood and then decreasing at older ages), as has been pointed out by multiple studies (*28*, *32*, *35*, *40*, *52*).

### Diet

There have been several metabolomic changes potentially attributable to diet. Perhaps the most commonly observed association is with caffeine (*26*, *37*, *57*), which tends to increase with age, including in the CSF (*51*). In the study of Lawton *et al.* (*26*), researchers found that while caffeine levels rose, the ratio of paraxanthine (the primary metabolite of caffeine in humans) to caffeine (paraxanthine:caffeine) decreased, a finding they suggested might indicate changes in the cytochrome P450 system. Beyond caffeine, several research groups have suggested that changes in creatine and creatinine (meat intake), TMAO (fish or salt intake), 2-hydroxybenzoate (fruit and vegetable intake), and citrate might be related to diet (*27*, *32*, *33*). The branched chain amino acids (BCAAs; leucine, isoleucine, and valine) are essential amino acids acquired through diet, and they are known to have a complex relationship with age and age-related phenotypes (*65*). In population studies, this complexity seems to appear with inconsistent directions of effect in the association of BCAAs with age. Valine was positively associated with age in women (*24*, *54*) and in centenarians/nonagenarians compared to typical elderly (*58*) but negatively associated with age in men (*50*). Isoleucine was also associated with age in inconsistent directions: studies have reported an increase in CSF (*53*), decrease in plasma (*40*, *50*), and increase in plasma (*24*, *34*).

### Oxidative stress

Oxidative stress has been another major theme in aging metabolomics (*26*, *30*, *33*, *36*, *39*, *41*, *46*, *50*, *54*, *55*). Some of the more consistently associated metabolites are carnitines, particularly acylcarnitines (*30*, *36*, *43*, *57*). Acylcarnitines tend to decrease at older ages, which may be related to the use of the carnitine-acylcarnitine shuttle in mitochondria to help mitigate oxidative stress. Carnitines more generally have been associated with age (*46*, *49*, *52*), with some studies reporting higher carnitine levels (*41*, *53*, *54*) and others lower levels (*25*, *36*, *43*). Glutathione, glutathione disulfide, ophthalmic acid (an analog of glutathione), and the glutathione/oxiglutathione ratio have also been found to decrease with age (*39*, *52*, *55*), suggesting that these antioxidant mechanisms may be compromised at older ages. Studies have also reported altered sphingolipid levels (*35*, *36*, *42*, *43*), particularly an increase in sphingomyelins (*30*, *34*), which might reflect processes related to the conversion of sphingomyelins to ceramides in relation to oxidative stress and inflammation. Other age-associated metabolites that have been interpreted as evidence of oxidative stress include carnosine and its precursor histidine (decreased with age) (*30*, *39*, *52*), eicosanoids (*33*), vitamin E (*46*), serine [both decreased (*50*) and increased (*41*)], glutamate (decreased) (*50*), and certain TCA cycle intermediates (increased) (*54*).

### Inflammation

Inflammation-related metabolites have also been observed to change with age. While many studies have linked their findings to inflammatory pathways (*26*, *33*, *35*, *41*, *45*, *46*, *50*, *51*, *55*), the metabolites in those associations have varied. The metabolites positively associated with aging include purine degradation compounds (*26*), complement protein peptides (*26*), ornithine (*41*), PUFAs (*45*), metabolites related to the cytochrome P450 system (*51*), and kynurenic acid (*55*). Metabolites that were negatively associated with aging include glutamate (*50*), although other studies have identified an increase in glutamate with age (*26*, *31*, *32*). Among presumably healthy-aging centenarians, studies have found changes in tryptophan (decreased) (*33*), lysophosphocholines (decreased) (*33*), eicosanoids (*33*), 2-hydroxybenzoate (increased) (*33*), phenylalanine (increased) (*35*), and acetylglycoproteins (increased) (*35*) in centenarians relative to other elderly or younger individuals.

## POPULATION-SPECIFIC AGING METABOLOMICS

While general changes in the metabolome with aging have been well studied, the differences in the aging metabolome within specific populations are less well understood. Given the many known influences on the metabolome [including sex, diet, exercise, and body mass index (BMI)] (*8*, *9*), it is reasonable to expect some interaction effects of these factors with age on metabolite levels, interactions that will be critical to understand for the sake of precision health applications. However, the differences in metabolomics technologies and study design between studies (Table 1) make direct comparison between studies on different populations harder to interpret. Most knowledge of these demographic and environmental interactions with aging metabolomics has come from a relatively small number of studies that have directly examined differences in age-metabolite associations between different groups.

### Sex

One of the most frequently studied influences on age-metabolite associations is sex (*24*, *26*, *27*, *34*, *37*, *40*, *42*, *53*, *56*, *57*), typically explored through stratified analyses (although these may be biased if the sample size is imbalanced) or direct interaction effects testing. One of the more frequently reported differences by sex is with menopause. For instance, in one large NMR study, VLDL, LDL, cholesterol, and TGs tended to increase with age for all participants overall, but among men, this increase was evident starting from the early 30s and onward, but women did not show similar trends until around age 50, presumably related to the timing of menopause (*34*). In another study (*42*), 68 significant age-sex interaction terms were identified among metabolites, including several for sphingolipids, phosphatidylcholines, and cholesterol, perhaps echoing similar interactions seen in a pair of earlier studies (*34*, *40*). Other reported interaction effects include more pronounced increases in urea and α-tocopherol in women than men (*26*); age-sex interactions for caffeine, cysteine, a vitamin D metabolite, and inositol (*37*); and a greater increase in 5-hydroxytryptophan with age in men than women (*53*).

### Race, ethnicity, and region

Interaction effects of age with race, ethnicity, or region have only been reported in a few studies. One of these studies was performed in the United States with a relatively diverse cohort (using the categories “Caucasian,” “African-American,” and “Hispanic”) (*26*). While numerous age-sex interaction effects were identified, no age-race/ethnicity effects were observed. Another study that examined differences by population group did so by comparing urine metabolomic profiles between American (United States) and Taiwanese participants (*32*). The age-metabolite associations between the populations had considerable overlap, including similar age associations noted for phenylacetylglutamine, 4-cresyl sulfate, HMB, and creatine. Some age-metabolite associations were unique by population, including associations seen only in the American population for *N*-methylnicotinamide, *N*-methylnicotinic acid, and *N*-methyl-4-pyridone-3-carboxamide. The relationship of these nicotinic acid metabolites with neurodegeneration and cognitive dysfunction provides a potential example of population differences in aging identified through metabolomics.

### Health status

Perhaps the most intriguing interaction effects have been from comparative studies by health status. Two studies investigated the metabolomic roots of longevity, with one study comparing children of nonagenarians to children of controls (*29*) and the other study comparing centenarians to typical elderly (around age 70) (*35*). In the former study, the children of nonagenarians tended to have more beneficial lipid profiles (larger LDL particles and lower TG levels) than the children of the controls. In the latter study, the centenarians showed numerous differences that might indicate better antioxidant and lipid remodeling capacities and less cellular senescence. Specifically, centenarians had higher phenylalanine (anti-inflammatory), higher sphingomyelins, and lower glycerophosphocholine (related to senescent cells). A study comparing younger adults, older exercisers, typical older individuals, and older individuals who were physically impaired identified a general trend in several key metabolites [including nicotinamide adenine dinucleotide (NAD^+^) and ophthalmic acid] that spanned the spectrum from younger adults to older exercisers to older and physically impaired, suggesting that aging-related changes might be modifiable through exercise (*55*).

## SAMPLE TYPE SPECIFICITY OF AGING METABOLOMICS

Unlike genomics, where an individual’s genetic sequence essentially remains the same throughout life, metabolomics is dynamic by time and location. Thus, the metabolomic profiles of different bodily fluids or tissues are likely to yield different biological information, making sample type an important consideration for study design and interpretation. Most large-scale human metabolomics studies of aging have been performed in plasma, serum, or urine samples, but the 2020s have ushered in greater diversity in sample type (Table 1). Several studies have now been performed in the CSF, yielding findings that might be of particular relevance to neurological conditions. For example, a study of CSF among individuals without neurodegeneration found themes of altered metabolites from the cytochrome P450 system, energy metabolism, the immune system, and γ-aminobutyric acid (GABA) (*51*), and another study of the CSF found an increase of 5-hydroxytryptophan, which is a precursor to serotonin (*53*). A study in children’s CSF found that 17 of the 30 metabolites they studied were associated with age, suggesting that an understanding of metabolomic changes with age is critical when assessing neurometabolic disease (*47*). A recent study of aging in saliva metabolites found several amino acids associated with age, including anserine and glutamate, which might be related to changes in taste sensory pathways in the elderly (*52*). In 2022, a study of aging metabolomics in muscle biopsies identified increases in dihydroxyacetone phosphate and 3-methoxytyramine with age, both of which are related to mitochondrial respiration and might indicate changes in the musculature (*55*). As the diversity of sample types grows, the insights into cell- and tissue-specific metabolic changes in aging will become more nuanced.

## THE CLINICAL IMPORT OF AGING METABOLOMIC PATHWAYS

The seven metabolomic pathways identified by aging studies detailed above—lipids and lipoproteins, steroid hormones and menopause, renal system and excretion, amino acids, diet, oxidative stress, and inflammation—represent not just a collection of markers of aging but a set of biological processes with important health ramifications. Lipoproteins such as VLDL, LDL, and HDL have an important role of transporting cholesterol (a lipid) throughout the body, and these lipoproteins have long been included in standard clinical assays evaluating dyslipidemia (*66*). Dysregulated lipid and lipoprotein levels (particularly high LDL and low HDL) have been associated with a variety of conditions, including coronary heart disease, ischemic heart disease, obesity, metabolic syndrome, type 2 diabetes, hypothyroidism, and chronic renal disease (*67*, *68*), which themselves impose an enormous burden of morbidity and mortality worldwide (*69*).

Steroid hormones affect a diverse array of physiological processes (*70*, *71*). Changing steroid hormone levels have been associated with numerous conditions such as ovarian cancer (*72*), frailty (*73*), lower urinary tract symptoms (*74*), inflammation (*75*), and Alzheimer’s disease (*76*), while menopause has been associated with cardiovascular disease risk (*77*) and osteoporosis (*78*, *79*).

With the renal system and excretion function, age-related changes in the kidney are commonly observed, including decreases in glomerular filtration rate, renal blood flow, and tubular capacity for secretion and reabsorption (*80*). Risk for chronic kidney disease increases with age (*81*) as does risk of progression to end-stage renal disease, which may require a kidney transplant or dialysis (*82*).

Changing amino acid levels may reflect changes to musculature with aging, which includes sarcopenia, a loss of muscle mass, which may be driven by decreased functional motor units, axonal withdrawal, decreased anabolic hormones and protein synthesis, or increased muscle catabolism (*83*). The clinical impact of this muscular loss can include greater frequency of falls, osteoporosis, obesity, arthritis, dyspnea, and joint instability, which all can result in worse quality of life and loss of independence (*83*, *84*). Some interventions have been explored that involve supplementation of specific amino acids, including BCAA (especially leucine) and HMB, that may potentially help recover muscle synthesis (*85*).

Beyond the fundamental importance of nutrition to general health and physiology, diet has been associated with aging and age-related diseases. In studies of longevity, one of the more intriguing interventions to improve lifespan is dietary or calorie restriction, since both the amount and makeup of food has been associated with survival in rodents, fruit flies (*Drosophila melanogaster*), and nematode worms (*Caenorhabditis elegans*) (*86*). In studies of dementia, dietary interventions such as the MIND diet (Mediterranean-DASH diet intervention for neurodegenerative delay) have shown potential for slowing the rate of cognitive decline (*87*).

Oxidative stress has historically been recognized as part of the biology of aging, including as one of the consequences of mitochondrial dysfunction, a proposed hallmark of aging that can lead to free radical generation, reactive oxygen species, and oxidative stress (*4*). Oxidative stress has been linked to the pathophysiology of many diseases, including atherosclerosis, chronic obstructive pulmonary disease, idiopathic pulmonary fibrosis, hypertension, type 2 diabetes, Alzheimer’s disease, cancer, and others (*88*).

Inflammation is another common feature of aging with diverse effects on health. The concept of inflammaging has been proposed as a way to describe the general and sustained increase in inflammatory markers such as C-reactive protein (CRP), interferons alpha and beta (IFN-α and IFN-β), tumor necrosis factor (TNF), several interleukins, and others as people age (*89*). Chronic inflammation has been linked to numerous health conditions, including cardiovascular disease, type 2 diabetes, chronic kidney disease, nonalcoholic fatty liver disease, autoimmune disorders, neurodegenerative disease, obesity, atherosclerosis, and asthma (*90*, *91*).

Together, these seven biological pathways identified among aging metabolomics studies have numerous consequences relevant to health and disease. The ability to track these changes at scale in human cohorts opens up new possibilities for diagnosis and prognosis for the diseases of older age.

## BRIDGING AGING METABOLISM AND METABOLOMIC FINDINGS

Metabolomics, as used here, refers to the study of the levels of small molecules in the body, particularly as measured by large-scale metabolomics platforms such as MS and NMR. Metabolism, however, is more encompassing and includes the study of all of the fundamental and essential chemical reactions of the body that break down, build up, and eliminate different compounds. While a fuller discussion of the biology of aging has been well covered in previous reviews (*4*, *92*–*95*), it is worth mentioning some of the major biological pathways of aging pertaining to metabolism and metabolic processes that have particular relevance to the findings from human population metabolomics studies.

One such pathway is nutrient sensing. Numerous studies of aging have implicated the insulin, insulin-like growth factor 1, mammalian target of rapamycin (mTOR), adenosine monophosphate–activated protein kinase (AMPK), and sirtuin pathways, which respond to levels of nutrients and represent potential points of intervention for longevity (*4*). Some sirtuins can be activated in the presence of NAD^+^, leading to increased mitochondrial activity (*96*). Among the human population studies reviewed above, NAD^+^ was associated with age in several studies. NAD^+^ was the main metabolite association identified in muscle cells (*55*), and it was also associated with age in both saliva (*52*) and RBCs (*39*). The observed changes in NAD^+^ in human cohorts is especially notable given the connection of NAD^+^ with aging (*97*) and the development of drugs to increase NAD^+^ levels (*98*). BCAAs also have roles in nutrient signaling pathways and have been implicated in aging research through caloric restriction, mTOR activity, and other mechanisms (*65*, *99*). Numerous population metabolomics studies have corroborated the association of BCAAs with aging, with associations noted for leucine (*39*), isoleucine (*24*, *34*, *39*, *40*, *50*, *53*), and valine (*24*, *50*, *54*).

Another major hallmark of aging is mitochondrial dysfunction along with altered TCA cycle and reactive oxygen species pathways. A number of elements contribute to this dysfunction, including mutations in mitochondrial DNA, reduced generation of mitochondria or removal of damaged mitochondria, destabilized electron transport chain complexes, and others, resulting in a reduction of the effectiveness of mitochondria in generating energy and leading to greater oxidative stress (*4*). As noted above, age has been associated with changing levels of numerous metabolites associated with oxidative stress, which might reflect aging-related problems with mitochondrial function. In addition, some studies have found altered levels of metabolites from the TCA cycle (*26*) [such as citrate (*43*, *54*)], which might also be indicative of mitochondrial dysfunction. Another metabolite relevant to the TCA cycle, acetyl–coenzyme A (CoA), has been linked to aging, connected to histone acetylation, gene expression, and sirtuins, among other mechanisms (*100*, *101*). The study of metabolites in RBCs identified an increase in pantothenate, a precursor of CoA, among elderly participants, which may indicate dysregulation of CoA synthesis and effects on downstream acetyl-CoA metabolism (*39*). FAs, which can be broken down to generate acetyl-CoA within mitochondria and which are themselves related to oxidative stress and aging (*102*), have also been associated with age in several metabolomic studies (*43*, *56*, *103*).

Methionine metabolism has also been implicated in aging, notably from studies of the beneficial effects of methionine restriction on longevity. The methionine cycle is related to the production of numerous other metabolites, including *S*-adenosylmethionine (SAM), polyamines (via the methionine salvage pathway), and cysteine, glutathione, and taurine (via the transsulfuration pathway) (*104*). In human metabolomics cohorts, methionine itself has been associated with age repeatedly, including in plasma (*40*, *43*), serum (*37*), and CSF (*53*). A decrease in arginine and an increase in ornithine was also observed in serum, both of which can feed into the polyamine synthesis pathway (*41*), and several ratios between serum levels of ornithine, arginine, putrescine, and spermidine were associated with age (*59*). Glutathione was observed to be associated with age (*39*, *52*, *55*), which might indicate aging-related changes involving the transsulfuration pathway.

## CHALLENGES OF THE FIELD

While much has been learned about the aging metabolome, there are many challenges to its study, some of which are inherent to contemporary metabolomics methodology. For instance, difficulty in compound identification often leads to unidentified metabolites that can obscure underlying themes in the results. Some metabolomics researchers have been successful in using multiple modalities (*40*) or integrating genetic association data (*105*) to help resolve unknown identities, but the issue is likely to persist as reference libraries, and identification methods continue to improve. Another challenge is that metabolite quantification is typically relative instead of absolute, which adds to the challenge of combining datasets from different studies. Similarly, comparing results across studies is made difficult by incomplete overlap in the metabolites measured. The growing use of more standardized commercial platforms has made comparison more feasible when the same platform is used, but cross-platform metabolite overlap among the more common groups (such as Metabolon, the Broad Institute, Biocrates, and Nightingale Health) may be low [<65% pairwise overlap according to a study from the COMETS consortium (*23*)].

**Fig. 3.**
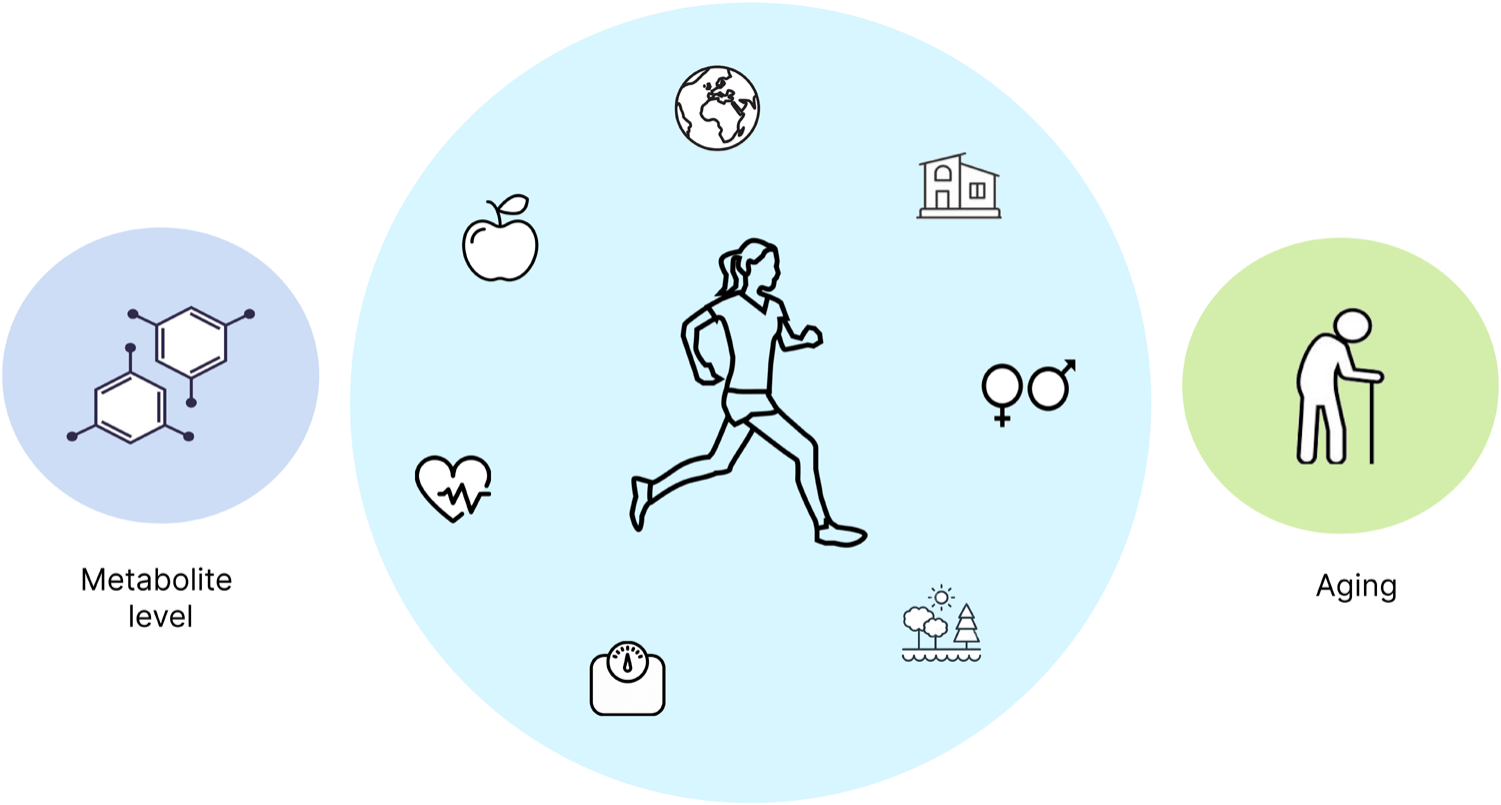
Complex interplay of aging and metabolism underneath population-level associations. The age-metabolite associations observed in population studies may reflect a number of underlying processes. One interpretation is that metabolic changes initiate or exacerbate aging processes or affect survival, leading to observed age-metabolite associations in the population. In the reverse conceptualization, biological changes occurring as part of aging processes lead to changes in metabolite level. In both cases, confounding and interaction effects with high-level demographic and lifestyle characteristics—including exercise, social determinants of health, sex, environment, BMI, health conditions, diet, and race and ethnicity—may be modifying the observed associations. Teasing apart these different effects from observational and often cross-sectional data is challenging, requiring careful study design and interpretation.

More specific to the field of aging metabolomics, there are a number of obstacles in study design and interpretation (Fig. 3). Perhaps the most difficult to resolve is the issue of confounding. Given how dynamic the metabolome is and how interconnected aging processes are with social, demographic, environmental, and lifestyle factors, there are many potential confounders. The factors most commonly controlled for in the aging metabolomics literature (and much of the human metabolomics literature at large) are sex and, to a lesser extent, BMI. However, some identified age-metabolite associations might be related to diet or health status, raising the question whether the associations are noncausal, causal, or reverse causal. One might imagine other factors confounding population metabolomics results, such as income, education, exercise, and chronic health conditions, although these elements are rarely controlled for. Regarding health status, many aging metabolome studies use “healthy” participants, but the word healthy is not always well defined nor is it necessarily the best choice for understanding the aging metabolome. As aging is a risk factor for many diseases, studying only individuals without disease, particularly at older ages, might be inducing a selection bias by studying only healthy agers. Nevertheless, with metabolomics data being added to large-scale population studies with a wealth of social, economic, nutritional, medical, and other data (*21*), we anticipate more studies exploring the role of potential confounders with the aging metabolome.

A related limitation is the lack of longitudinal data collection (*63*, *106*, *107*). Most studies of the aging metabolome have been performed cross-sectionally, with only one sample per person, and many earlier studies grouped participants into age bins (usually, younger or middle-aged adults compared to the elderly). The few studies that included longitudinal samples have often only had a handful of samples from participants over a few years (*42*, *44*) or at two different time points spaced further apart (*41*). Nearly all studies have been in adults as well despite much variation being seen with age among children (*47*). With a sizeable portion of metabolite variability driven by “unstable” day-to-day variation (*108*), the lack of high-resolution longitudinal data might be obscuring insight into the changes of the metabolome with age. Longitudinal data on a larger scale would also be useful in disentangling cause and effect. For instance, while a theme of altered metabolites related to oxidative stress is evident from multiple studies, the causal direction of these relationships is unclear, as an elevated metabolite might be causing oxidative stress, caused by some upstream process that separately causes the change in metabolite and oxidative stress or caused by the oxidative stress. While prior biological knowledge may provide reasonable clues to the role a given metabolite plays, novel discoveries of metabolite associations will require additional follow-up to determine the appropriate causal relationships.

## OPPORTUNITIES MOVING FORWARD

**Fig. 4.**
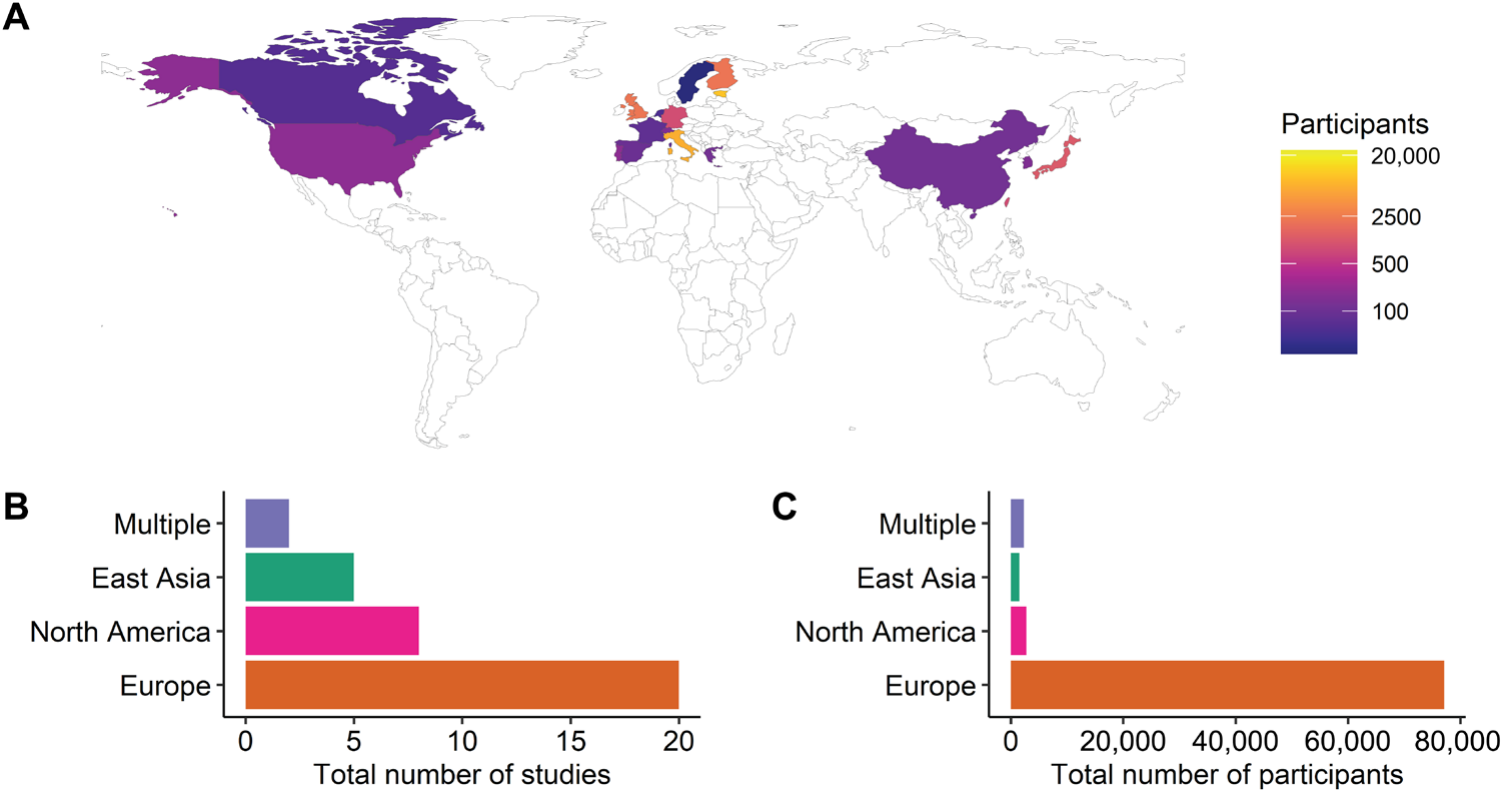
Distribution of aging metabolomics population studies worldwide. (**A**) The distribution of participants in the reviewed aging metabolomics studies by country is shown. Most studies have been located in North America, Europe, or East Asia, leaving a substantial gap in our understanding of aging across the world. (**B**) The regional breakdown of aging metabolomics human population studies by study count and (**C**) participant count is shown. In both cases, Europe has been represented to a greater extent than other regions. The category “Multiple” includes studies that had cohorts from more than one of the regions listed. Transparent regions were not represented among the human aging metabolomics studies reviewed here.

Moving forward, there are many new avenues of research to explore. Perhaps the most evident need is for increased diversity of aging metabolomics research in several respects. Regarding regional, racial, and ethnic diversity, of the 36 studies reviewed here, 29 (81%) were based in North America or Europe, and 6 (17%) were based in East Asia, leaving much of the world underrepresented (Fig. 4). Even for the geographic regions where many studies have been performed, not all populations within those regions have necessarily been well represented. Especially given the differences in experiences of aging across the world, it is critical that a more diverse set of populations be included in future work. Similarly, since age-associated metabolites presumably provide insight into the typical functioning of the human body, we would benefit from more studies of the metabolome among children and young adults. The study of children reviewed here (*47*) identified nonlinear relationships of metabolites with age, pointing to complex developmental influences on metabolite levels that might provide fundamental insight into metabolic regulation. Furthermore, a greater understanding of the changing metabolome in children would likely aid efforts to identify, diagnose, and treat childhood metabolic conditions using increasingly accessible metabolomics technology that may eventually be commonly used in clinical applications. In addition, as mentioned above, the metabolome is highly context specific, so continuing to increase the diversity of sample types explored will likely yield fruitful results.

On a more methodological note, one opportunity is to explore more nonlinear effects of aging (*106*, *107*). Most studies have used some form of linear analysis to identify metabolites changing with age, yet the nonlinear trajectories seen among children (*51*) and the shift seen with menopause (*40*) both suggest that more nuanced models might be more appropriate, especially as wider age ranges or dense longitudinal sampling are included. Indeed, nonlinear models have already been useful in metabolomic clocks to predict chronological age (*109*). Machine-learning methods and deep-learning (particularly neural network–based methods) can be well suited to handle complex relationships and have been increasingly used in metabolomics work, which has been well described in several recent reviews (*110*–*114*). The impact of these methods on the aging metabolomics field can be seen already through the use of fuzzy c-means clustering to identify aging trajectories (*45*) or the use of support vector machines, regularized regression, and partial least squares (PLS) methods to predict age (*40*).

Tools from the network analysis literature will also be helpful in understanding the role of the metabolome in age. Many studies have used pathway enrichment analysis as a means of identifying overrepresented pathways among sets of associated metabolites, such as the mummichog method (*115*), which has been used in several aging metabolomics studies (*46*, *49*). Other approaches focus on network inference or topology to study the properties of metabolic networks, similar to what has been used with other omics data, such as gene coexpression and protein interaction networks (*116*, *117*).

While metabolomics alone can provide a wealth of information, yet greater insights can be achieved through integration with other omics datasets. Some of the early work on omic clocks of aging was driven by epigenetic data (*118*), and more recent omic clocks have included the transcriptome, proteome, and telomeres (*119*, *120*). Multiomic analysis has been useful already in identifying different patterns of aging (*44*), which suggests that our understanding of the aging metabolome would benefit from jointly generated data on other omes that could help contextualize the metabolic changes that are occurring. The best way to integrate different kinds of omics data is an active area of research that has been the subject of several excellent reviews (*121*, *122*). Several methods exist that leverage the growing metabolite genome-wide association study (GWAS) datasets (*123*) to study metabolites in novel ways. For instance, the popular transcriptome-wide association study (TWAS) framework that uses genomic data to impute transcript levels in order to detect gene-phenotype associations (*124*, *125*) has since been extended to the imputation of metabolomic data (*126*). Another method, Mendelian randomization (MR) (*127*), uses genetic mutations as an instrumental variable to estimate the effect of one phenotype on another (given that the assumptions of MR are met) and has similarly been extended to the study of metabolite-phenotype relationships (*128*).

A number of methods rooted in systems biology have provided a different mode of integrating other kinds of data into metabolomics analysis. The field of fluxomics focuses more on the dynamic changes in the flux through a metabolic network, using tracer-based experimental data, reaction kinetics, thermodynamics, stoichiometry, and other information about the underlying metabolic network in a variety of ways (*129*–*132*). These holistic approaches have been useful in many contexts, including identifying new metabolic and regulatory functions and finding potential drug targets (*131*). Another systems biology approach combined blood metabolomics data with transcriptomics data, mapped them to the Human Recon 2 metabolic network, and compared the network-based distance of metabolites and transcripts to their correlation in blood, which allowed them to evaluate the kinds of proteins typically correlated with blood metabolite levels (*133*). The application of these and other systems biology approaches will undoubtedly be useful to the study of aging in its full complexity.

## THE FUTURE OF AGING, METABOLOMICS, AND PRECISION HEALTH

One of the goals in medicine is precision health: the incorporation of individual-level characteristics (genetics, physiology, demographics, and environment) into the management of one’s health. The multiomic technology revolution that has made deep molecular profiling possible has also opened the doors to personal physiological tracking (*134*). These data hold tremendous promise for expanding our understanding of biology and disease etiology, but the proper contextualization of this multiomic landscape—at both the individual and population level—is a necessary precursor to precision health applications. Since aging is the largest risk factor for mortality and one of the most important risk factors for chronic diseases, understanding the omics of aging processes will be crucial. As reviewed above, several studies have begun exploring the diversity of aging experiences, from nonagenarians and centenarians (*29*, *33*, *35*) to healthy agers (*43*) and ageotypes (*44*). This knowledge will help provide the backdrop against which precision aging data are interpreted as we seek to understand an individual’s personal health trajectory. The success of precision health in aging will depend, in no small part, on how comprehensively human metabolomics cohorts are able to study aging across the spectrum of populations, sample types, and conditions: the diversity of research will drive the universality of application.

Aging is a complex phenomenon that is difficult to define, and its many connections with disease represent a challenge for causal inference. Yet disentangling the molecular drivers of these aging processes will be key to managing the disease risk they confer. The growing field of population aging metabolomics offers tremendous promise for understanding aging and how it varies over time and context in humans, which will be crucial to building a foundation for precision health in an increasingly aging world.
